# Polystyrene Nano- and Microplastic Particles Induce an Inflammatory Gene Expression Profile in Rat Neural Stem Cell-Derived Astrocytes In Vitro

**DOI:** 10.3390/nano14050429

**Published:** 2024-02-27

**Authors:** Kristen A. Marcellus, Steven Bugiel, Andrée Nunnikhoven, Ivan Curran, Santokh S. Gill

**Affiliations:** Regulatory Toxicology Research Division, Bureau of Chemical Safety, Health Products and Food Branch, Health Canada, Ottawa, ON K1A 0K9, Canada

**Keywords:** microplastics, nanoplastics, transcriptional changes, inflammation, stem cells, reactive astrocytes, astrogliosis

## Abstract

Microplastics are considered an emerging environmental pollutant due to their ubiquitous presence in the environment. However, the potential impact of microplastics on human health warrants further research. Recent studies have reported neurobehavioral and neurotoxic effects in marine and rodent models; however, their impact on the underlying cellular physiology in mammals remains unclear. Herein, we exposed neural stem cells and neural stem cell-derived astrocytes, oligodendrocytes, and neurons to various sizes and concentrations of polystyrene nano- and microplastics. We investigated their cellular uptake, impact on cytotoxicity, and alteration of gene expression through transcriptome profiling. The cell type most affected by decreased viability were astrocytes after 7 days of repeated exposure. Transcriptional analysis showed that 1274 genes were differentially expressed in astrocytes exposed to 500 nm microplastics, but only 531 genes were altered in astrocytes exposed to 50 nm nanoplastics. Both canonical pathway and Kyoto Encyclopedia of Genes and Genomes analysis showed that upregulated pathways were involved in neuroinflammation, innate and adaptive immunity, cell migration, proliferation, extracellular matrix remodeling, and cytoskeleton structures. The downregulated pathways were involved in lipid metabolism, specifically fatty acid oxidation and cholesterol metabolism. Our results show that neural stem cell-derived astrocytes repeatedly exposed to nano- and microplastics for 7 days undergo changes that are hallmarks of astrogliosis.

## 1. Introduction

Plastic is considered an emerging, persistent environmental pollutant and plastic particles have been detected in various food commodities [1,2,3]. Global plastic production reached between 368 and 460 million tonnes in 2019 and 1.5–4.0% is released annually into the marine environment [2,4,5]. Exposure of plastic debris to environmental conditions causes plastics to further disintegrate into smaller microplastics (<5 mm, MPs) and nanoplastics (<100 nm, NPs) by the processes of photo- and thermo-oxidative degradation, biodegradation, mechanical fragmentation, and hydrolysis [2,6,7,8]. Micro- and nanoplastics (MNPLs) can be categorized into two groups: primary MNPLs are intentionally manufactured at the micro- and nano-sized scale for industrial, agricultural, biomedical and domestic use, whereas secondary MNPLs are not intentionally produced, but are the result of degradation and fragmentation of larger plastic waste [8,9]. Plastic particles have been found in the most remote, secluded, and pristine regions of the planet [10,11].

Various types of MNPLs, composed of different types of polymers such as polyethylene (PE), polystyrene (PS), polyvinylchloride (PVC), polyethylene terephthalate (PET), and polypropylene (PP), have been found in different environmental matrices across ecosystems, including oceans, freshwater, sediments, and atmospheric fallout [1,2,3,9,12]. Due to the pervasive presence of MNPLs in the environment, including water and air, human exposure may occur via ingestion or inhalation [8,13,14]. The presence of MPs have been reported in various foods, such as seafood, seaweed, bottled and tap water, milk, honey, sugar, and salt [9,15,16,17,18]. Furthermore, MPs have been detected in human infant and adult stool samples (20 particles/10 g stool) [19,20], as well as placenta and lung tissue [21,22]; however, the potential impact of MNPLs on human health requires further research.

Several in vitro and in vivo studies have revealed that MNPLs enter various cells and organs/tissues, respectively. In vivo studies have shown, that once in the body, MNPLs can circulate to deposit in various organs, such as lungs, stomach, liver, kidney, and brain [17,23,24]. Following oral ingestion, it is estimated that >90% of particles remain in the gut lumen and are excreted, and therefore, only a limited number of particles are transported into the gut and further into systemic circulation via microfold cells in the Peyer’s patch and paracellular persorption [25,26,27]. The size range of MNPLs, especially <150 µm, makes it possible for them to cross through an organism’s barriers [1,17,28,29,30,31]. In particular, smaller sized particles (0.1–10 µm) may cross the blood–brain barrier and the placenta [25,32]. Through these exposures, MNPLs not only cause physical and mechanical damage, but also induce oxidative damage, gut dysbiosis, intestinal barrier dysfunction, neurotoxicity, liver metabolic disorder, kidney injury, an immune response, energy-related changes and altered gene expression [12,24,28,33,34,35,36].

Several studies have observed accumulation of MNPLs in fish and rat brain tissues, suggesting that these particles may cross the blood–brain barrier (BBB) [30,37,38,39]. Furthermore, several studies have documented the presence of MNPLs in the mammalian brain upon exposure to plastic particles, resulting in changes in synaptic plasticity, neuroinflammation, learning and memory impairment, decreased acetylcholine levels, alteration in energy metabolism, mitochondrial dysfunction, and dysregulated proteostasis [3,17,30,39,40]. Evidence indicates an association between exposure to MNPLs and neurobehavioral and neurodevelopment effects; however, the mode of action remains to be elucidated [3].

The brain is one of the most complex organs in the body and it comprises multiple cell types including neurons, astrocytes, oligodendrocytes, microglia and endothelial cells and their assortment of subtypes [17,41]. These cell types are derived from the differentiation of multipotent neural stem cells [42,43,44]. Each cell type has different functions that are highly adapted to local circuitries [42,44,45]. Astrocytes are the most abundant cell in the central nervous system (CNS) and their subtypes have diverse function and morphology, including assisting and supporting neurons, controlling the formation, maintenance, function, and removal of neuronal synapses, controlling blood flow and helping to regulate the BBB [46,47,48,49]. They are also involved in the control of immune cell activation and trafficking by detecting danger signals and secreting cytokines and chemokines [49,50]. More recently, Liang et al. (2022) noted cell-specific responses to nanoplastics in the brain, shedding light on the possibility of differing cell responses across neural cells [17]. Thus, it is important to not only investigate the impact of MNPLs on the brain, but on each individual cell type as well.

In this study we examined the different cellular responses to MNPLs and their toxicity in the three major cell types of the CNS: astrocytes, oligodendrocytes, and neurons, as well as their precursor neural stem cells (NSCs). Polystyrene (PS) latex beads with 50 nm (NPs) and 500 nm (MPs) diameters at a concentration of 0–1000 µg/mL were used to assess cell viability, cellular internalization of the PS-beads, and changes in the transcriptome. We demonstrate that of the cells we tested, astrocytes are the most sensitive to PS-MNPL cytotoxicity and that repeated exposure (7 days) induces a higher degree of cytotoxicity compared to 24 h exposure. Additionally, astrocytes and NSC were found to readily take up both sizes of PS-beads. Furthermore, transcriptomic analysis revealed an inflammatory gene expression profile in astrocytes following repeated exposure to MNPLs for 7 days, resulting in reactive astrocytosis or astrogliosis.

## 2. Materials and Methods

### 2.1. Polystyrene Microspheres

The polystyrene microspheres (Fluoresbrite^®^ YG and Polybead^®^) were obtained from Polysciences, Inc. (Warrington, PA, USA). The 50 nm spheres (17149-10; 08691-10) were packaged in 2.5% (*w*/*v*) aqueous suspension at a concentration of 3.64 × 10^14^ particles/mL. These beads have a coefficient of variation of 15%. The 500 nm spheres (17152-10; 07307-15) were also packaged in 2.5% (*w*/*v*) aqueous suspension at a concentration of 3.64 × 10^11^ particles/mL and have a coefficient of variation of 3%. In order to disperse the microspheres, the suspensions were vortexed for 30 s and diluted in complete media at final concentrations of 0.01–1000 µg/mL, where microbeads were evenly dispersed (Supplementary Materials Table S3).

### 2.2. Cell Culture

Rat fetal neural stem cells, tissue culture media and supplements were purchased from ThermoFisher (Waltham, MA, USA) unless stated otherwise. Rat fetal neural stem cells (NSCs) were cultured in KnockOut DMEM/F12 supplemented with StemPro NSC SFM (2%), b-FGF and EGF (20 ng/mL) (Peprotech, Cranbury, NJ, USA), GlutaMAX-1 (2 mM) and 1% penicillin/streptomycin. These cells were cultured on poly-l-ornithine- (MilliporeSigma, Oakville, ON, Canada) or Geltrex-coated (ThermoFisher) culture dishes according to manufacturers’ instructions. Rat neural stem cells were differentiated into astrocytes, oligodendrocytes, and neurons over the course of 7 days with specialized differentiation media and the appropriate coating matrix, according to manufacturers’ instructions, as previously used in our lab [51]. Briefly, NSCs at passage 2–3 were plated on poly-l-ornithine- and laminin-coated plates (Corning, Corning, NY, USA) and differentiated into neurons with neural differentiation medium consisting of Neurobasal Medium with 2% B-27 serum-free supplement and 2 mM GlutaMAX-1 supplement. NSCs were differentiated into astrocytes with differentiation medium consisting of D-MEM Medium with N-2 supplement (1%), 2 mM GlutaMAX-1 supplement and 1% FBS, while plated on Geltrex-coated plates. Oligodendrocyte differentiation medium consisting of Neurobasal Medium with 2% B-27 serum-free supplement, 2 mM GlutaMAX-1 supplement and T3 (30 ng/mL) (MilliporeSigma) was used to differentiate NSCs plated on poly-l-ornithine and laminin-coated plates into oligodendrocytes. The 24 h cultures were seeded at 8 × 10^3^ cells/well for NSC and 7 × 10^3^ cells/well for the differentiated cells on the appropriately coated 96 well plate. In the 7-day cultures, only the NSC and astrocytes were seeded at 3 × 10^3^ cells/well. Differentiation protocol was confirmed by immunostaining with cell specific markers (data not included). Cultures were routinely checked for mycoplasma using the MycoAlert^®^ Mycoplasma Detection Kit (Lonza, Morristown, NJ, USA). All cell cultures were incubated at 37 °C, 5% CO_2_.

### 2.3. Cytotoxicity Assay

CyQuant™ MTT Cell Viability Assay (ThermoFisher) was used according to the manufacturers’ quick protocol instructions. Cells were plated in black 96-well plates (Corning or ThermoFisher). NSCs were differentiated for 7 days prior to treatment with MNPLs. Cells were treated with non-fluorescent microspheres (Polybead^®^) 0–1000 µg/mL for 24 h or 7 days, with new microbeads in fresh media every 2–3 days. Absorbance was measured at 540 nm (Cytation 3; BioTek, Winooski, VT, USA). Viability of the control cells (not exposed to microspheres) was set to 100%.

### 2.4. Flow Cytometry

Neural stem cells were plated on 6 or 12 well plates (Greiner Bio-One, Monroe, NC, USA) and subsequently astrocytes were differentiated from NSC for 7 days prior to MNPL treatment. Cells were exposed to fluorescent (Fluoresbrite^®^ YG) microspheres for 24 h. Cells were washed with PBS and collected using accutase (ThermoFisher) at 37 °C. Cells were resuspended in staining buffer (5% FBS in PBS) with SYTOX™ blue dead cell stain (ThermoFisher) and analyzed on the BD LSRFortessa™ flow cytometer. Data analysis was performed using FlowJo™ v10.8 software (BD Life Science, Mississauga, ON, Canada).

### 2.5. Immunofluorescence

Cells were plated on 8 chambered slides (ThermoFisher) and treated with MNPLs for 24 h after 7 days of differentiation. Cells were washed 3× with PBS before being fixed with 4% formaldehyde in PBS pH 7.4 for 15 min and then permeabilized with 0.5% Triton X-100 in 1× PBS for 15 min. Cells were then blocked with 10% fetal bovine serum (FBS) for 2 h at room temperature. NSCs and astrocytes were incubated overnight at 4 °C with primary antibodies α-Nestin (1:200; Novus Biologicals, Toronto, ON, Canada) and α-GFAP (1:500; Cell Signaling, Danvers, MA, USA), respectively. Cells were washed with PBS and then incubated with Alexa Fluor™ secondary antibodies (1:500, GαChicken (Nestin) Alexa 594 Abcam, Boston, MA, USA) (1:500, GαRabbit (GFAP) Alexa 568 ThermoFisher) diluted in 1% FBS, 0.1% Triton X-100 for 2 h at room temperature. Cells were washed 3× with PBS and mounted in ProLong Diamond Antifade mounting media containing DAPI for staining nuclei (ThermoFisher). Cells were visualized by microscopy at 40× using the Cytation C10 (Agilent Technologies, Santa Clara, CA, USA) microscope.

### 2.6. RNA Extraction

RNA extraction was performed on astrocytes exposed to MNPLs for 7 days using the miRNeasy Mini kit for an *n* = 4 (Qiagen, Toronto, ON, Canada). RNA concentration, purity, and integrity were analyzed using the RNA 6000 Nano kit on the Bioanalyzer and Nanodrop (Agilent Technologies). The RNA integrity number was greater than 9 for all samples.

### 2.7. RNA Sequencing

Libraries were prepared in accordance with the Illumina Stranded mRNA Sample Preparation Guide (Illumina, San Diego, CA, USA). The resulting libraries were quantified using the Qubit 3.0 Fluorometer—Broad Range dsDNA assay (ThermoFisher) and validated using the Agilent 2100 Bioanalyzer DNA 1000 chip (Agilent Technologies). Libraries were normalized to 4 nM, pooled and subsequently loaded at a concentration of 1.9 pM onto a NextSeq^®^ 500/550 High Output Kit v2.5 (150 cycles) flow cell (Illumina).

RNA-sequencing reads were filtered out and trimmed using bbduk (https://jgi.doe.gov/data-and-tools/bbtools/, accessed on 10 November 2021) using the cutoffs of read length of at least 20 bases, and a quality score of at least 12. Filtered reads were aligned to the *Rattus norvegicus* genome (from Ensembl, version 6.0.90) using STAR [52] and featureCounts was used to calculate total read counts mapping to each gene. Reads with an alignment quality below 12 were disregarded. DESeq2 was used to calculate DEGs at each nanoplastic (50 nm) and microplastic (500 nm) concentration relative to the control (0 µg/mL) [53] and shrinkage estimators were calculated using apeglm. Negative binomial *p*-values were adjusted for multiple tests by DESeq2 using the Benjamini–Hochberg method; and for downstream analysis, the expression values used were variance-stabilized using the regularized log transform in DESeq2. Raw datasets can be accessed through GSE256038 for the NP 50 nm and MP 500 nm RNA-sequencing datasets.

### 2.8. Pathways Analysis

DEGs were analyzed using Qiagen Ingenuity Pathway Analysis (IPA) (Qiagen Inc., Hilden, Germany, https://digitalinsights.qiagen.com/IPA, accessed on 27 April 2022) to generate a list of both predictive activated (z-score ≥ 2) and inhibited (z-score ≤ −2) canonical pathways and upstream regulators. Gene Ontology (GO) Terms and Kyoto Encyclopedia of Genes and Genomes (KEGG) Pathway analyses were performed using the Database for Annotation, Visualization, and Integrated Discovery (DAVID) [54,55]. Upregulated and downregulated genes were analyzed through DAVID independently as separate data sets.

### 2.9. Statistical Analysis

All in vitro experiments were performed with 3 biological replicates. Data were presented as mean ± standard error of the mean (SEM). Data were analyzed with one-way analysis of variance (ANOVA) followed by Dunnett’s multiple comparison post hoc test in GraphPad Software v9 (Dotmatics, Boston, MA, USA). Significance was set at *p* ≤ 0.05.

## 3. Results

### 3.1. Cytotoxicity

**Figure 1:**
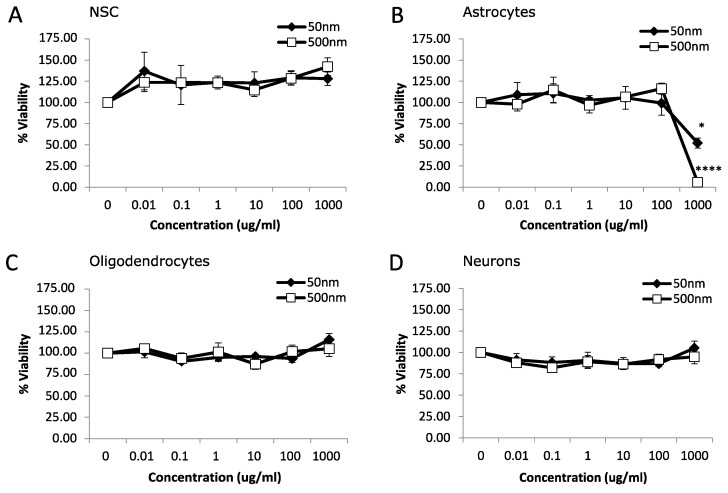
Viability after 24 h exposure to polystyrene particles. Cell viability was measured in neural cells exposed to polystyrene particles (50 nm and 500 nm) at concentrations of 0–1000 µg/mL for 24 h. Cell viability was assessed by MTT assay. Quantification of cell viability as a percentage relative to CTL (control, unexposed cells). (**A**) Neural stem cells (NSC). (**B**) Astrocytes. (**C**) Oligodendrocytes. (**D**) Neurons. Data is mean ± SEM from three independent experiments. One-way ANOVA with Dunnet’s post hoc test * *p* < 0.05, **** *p* < 0.0001.

We used rat fetal neural stem cells (rNSCs) and rNSC-derived oligodendrocytes, astrocytes, and neurons to investigate the potential toxicity of MNPLs (50 nm and 500 nm) on neural cells. An MTT assay was used to assess the viability of cells after 24 h or 7 d exposure to non-fluorescent PS-beads (0, 0.01, 0.1, 1, 10, 100 and 1000 µg/mL). After 24 h exposure, astrocytes displayed cytotoxicity with a 48% and 94% reduction in cell viability at the highest concentration, 1000 µg/mL, for both 50 nm and 500 nm PS-bead sizes, respectively (Figure 1B; *p*-value < 0.05). NSC, oligodendrocytes, and neurons had no significant differences (Figure 1A,C,D; *p*-value < 0.05).

**Figure 2:**
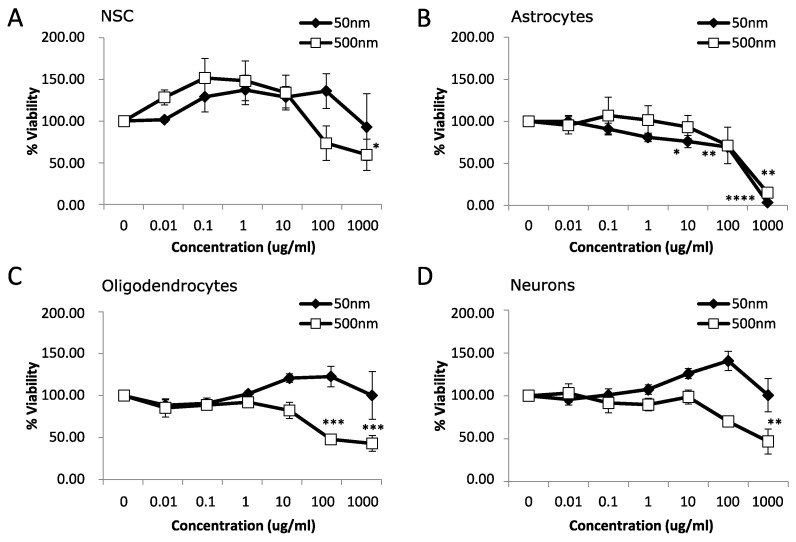
Viability after 7 days exposure to polystyrene particles. Cell viability was measured in neural cells exposed to polystyrene particles (50 nm and 500 nm) at concentrations of 0–1000 µg/mL for 7 days. Cell viability was assessed by MTT assay. Quantification of cell viability as a percentage relative to CTL (unexposed cells). (**A**) Neural stem cells (NSC). (**B**) Astrocytes. (**C**) Oligodendrocytes. (**D**) Neurons. Data is mean ± SEM from three independent experiments. One-way ANOVA with Dunnet’s post hoc test * *p* < 0.05, ** *p* < 0.01, *** *p* < 0.001, **** *p* < 0.0001.

After 7 days of MNPL exposure, NSC and neurons displayed mild cytotoxicity with a ~40–50% reduction in cell viability only with the 500 nm particles at 1000 µg/mL (Figure 2A,D; *p*-value < 0.05). Oligodendrocytes exhibited a more pronounced decrease in cell viability of >50% with the 500 nm particles at 100 and 1000 µg/mL (Figure 2C; *p*-value < 0.05). Astrocytes showed the most pronounced effect with decreased cell viability occurring at 10 µg/mL and continuing to >90% decreased cell viability at 1000 µg/mL with 50 nm particles. The 500 nm particles significantly decreased cell viability in the astrocytes by ~85% at 1000 µg/mL and showed a trend of ~30% decrease at 100 µg/mL (Figure 2B, *p*-value < 0.05). Subsequently, astrocytes were chosen for further study due to the increased cytotoxicity observed at both 24 h and 7 d exposure to both MNPLs.

### 3.2. Cellular Uptake

**Figure 3:**
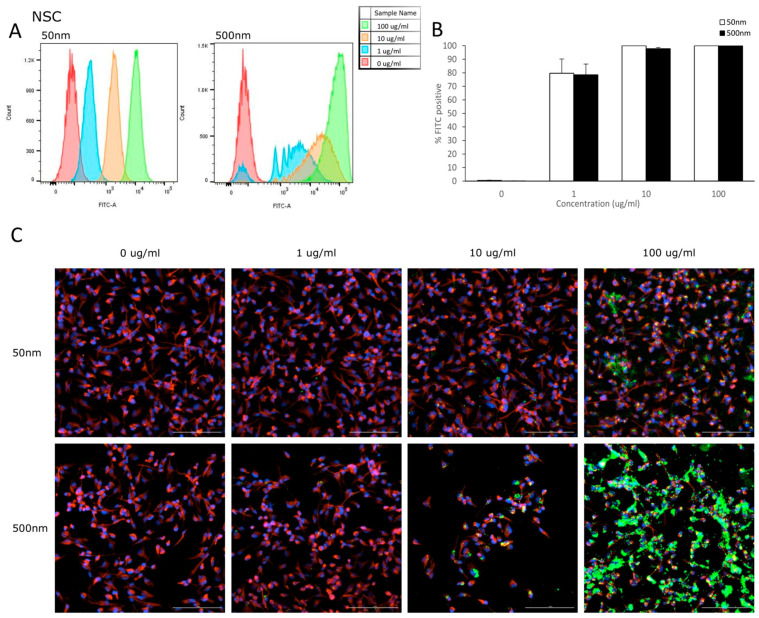
NSCs internalize PS-NPs and PS-MPs. Uptake was analyzed by flow cytometry after cells were exposed to YG-PS-beads for 24 h. (**A**) Representation of mean fluorescent intensity peaks of NSC exposed to YG-PS-beads. (**B**) Quantification of the percentage of NSCs that internalized YG-PS-NPs and -MPs (*n* = 3). (**C**) Representative images of NSC cells exposed to 50 nm and 500 nm YG-PS-beads for 24 h. Scale bar = 100 µm.

**Figure 4:**
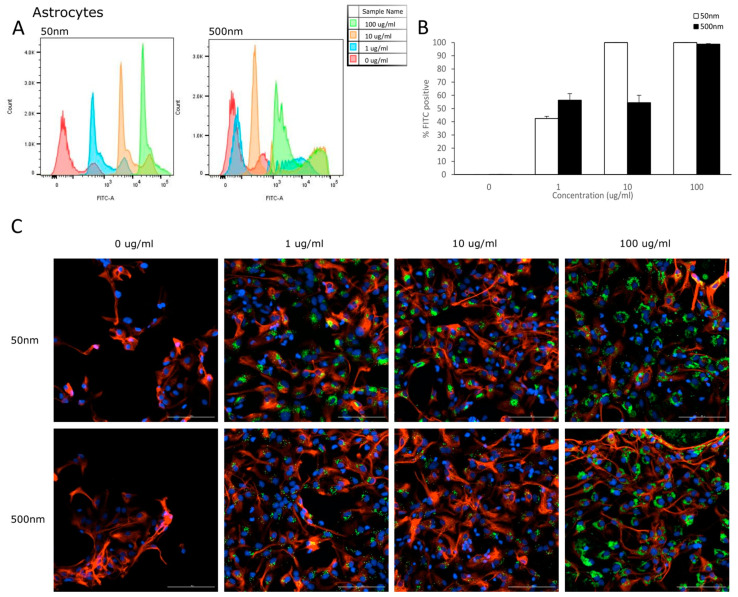
Astrocytes internalize PS-NPs and PS-MPs. Uptake was analyzed by flow cytometry after cells were exposed to YG-PS-beads for 24 h. (**A**) Representation of mean fluorescent intensity peaks of astrocytes exposed to YG-PS-beads. (**B**) Quantification of the percentage of astrocytes that internalized YG-PS-NPs and -MPs (*n* = 3). (**C**) Representative images of astrocyte cells exposed to 50 nm and 500 nm YG-PS-beads for 24 h. Scale bar = 100 µm.

Flow cytometry was used to analyze the uptake of PS-particles in astrocytes and their parental cell line (NSC). The fluorescence of each cell was assessed after exposure to YG-PS-beads (0, 1, 10 and 100 µg/mL) for 24 h. Unexposed cells were used to set the threshold of the fluorescent signal. Both NSCs and astrocyte cells showed internalization of PS-NPs and -MPs at all concentrations tested (Figure 3A and Figure 4A). In NSC cells, the percentage of cells that took up 50 nm PS-NPs was 79.7% for 1 µg/mL and saturation (100%) was reached by 10 µg/mL (Figure 3B). Meanwhile, 500 nm PS-MPs were taken up in 78.5%, 97.8% and 99.9% of cells at 1, 10 and 100 µg/mL, respectively (Figure 3B). Similarly in astrocytes, 42.5% of cells took up 50 nm nano-particles at 1 µg/mL and 100% for both 10 and 100 µg/mL (Figure 4B); while 500 nm micro-particles were taken up in 56.3% of cells at 1 µg/mL, 54.5% at 10 µg/mL and 98.8% at 100 µg/mL (Figure 4B). Cellular uptake was visualized using microscopy (Figure 3C and Figure 4C).

### 3.3. Differentially Expressed Genes (DEGs)

**Figure 5:**
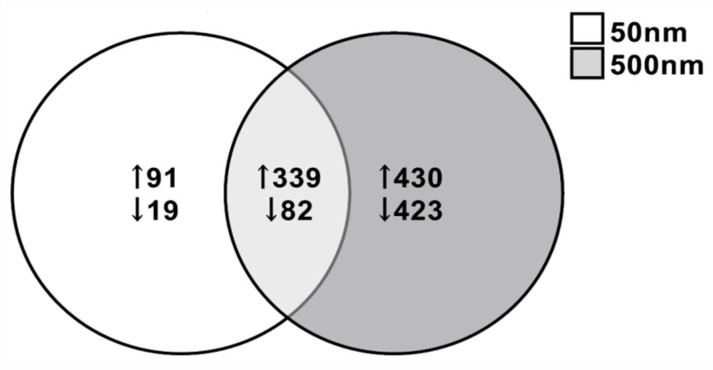
Venn diagram illustrating different and common differential gene expression in astrocytes after exposure to 50 nm and 500 nm PS particles for 7 days at 100 µg/mL (*n* = 4). A total of 39.4% of DEGs upregulated were common between both groups of astrocytes and 15.6% of DEGs were commonly downregulated between astrocyte groups. DEGs: ≤−1.5, ≥1.5; *p* < 0.05.

Due to the increased susceptibility of the astrocytes at both 50 nm and 500 nm PS exposure, we performed RNA-seq on astrocytes exposed for 7 days at 0, 1, 10 and 100 µg/mL. DEGs were considered significant at a fold change ≤−1.5, ≥+1.5 and *p*-value < 0.05. DEGs were most extensively observed at the highest dose tested (i.e., 100 µg/mL) and for this reason, we selected this dose for further analysis. A total of 531 DEGs were identified when comparing between 50 nm exposed astrocytes and control astrocytes, with 101 downregulated (19.0%) and 430 upregulated (80.97%) genes (Figure 5). Although we only saw a decreasing trend in cell viability in astrocytes exposed to 100 µg/mL of 500 nm PS particles, a total of 1274 DEGs were observed, 505 (39.64%) downregulated and 769 (60.36%) upregulated (Figure 5). Between the 50 nm and 500 nm exposed groups, 339 genes were commonly upregulated and 82 were commonly downregulated (Figure 5). The top 10 upregulated and downregulated genes in PS-MNPL treated astrocytes are reflected in Table 1 and Table 2, respectively.

**Figure 6:**
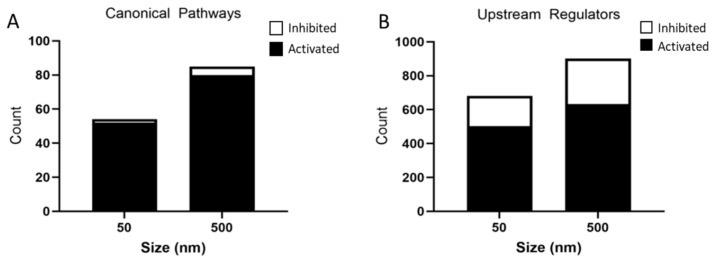
Analysis of significantly predicted activated and inhibited canonical pathways (**A**) and upstream regulators (**B**) in astrocytes exposed to 50 nm and 500 nm PS particles using IPA. Astrocytes exposed to 50 nm PS-NPs had 52 activated pathways and 2 inhibited pathways. Astrocytes exposed to 500 nm PS-MPs had 80 activated pathways and 5 inhibited pathways based on a Z-score < −2 and >2 and *p*-value < 0.05. The 50 nm PS-NP exposed astrocytes had 503 predicted activated and 177 predicted inhibited upstream regulators, while 500 nm PS-MP exposed astrocytes had 633 predicted activated and 269 predicted inhibited upstream regulators based on a Z-score > −2 and >2 and *p*-value < 0.05.

These DEGs were further analyzed by IPA and a list of canonical pathways and upstream regulators predicted to be affected by particle size were generated. The 50 nm exposed group had 52 activated pathways and 2 inhibited pathways, whereas the 500 nm exposed group had 80 activated pathways and 5 inhibited pathways based on a Z-score < −2 and >2 and *p*-value < 0.05 (Figure 6A). A top 10 list, where possible, of activated and inhibited canonical pathways was created for both the 50 nm and 500 nm exposed groups (Table 3 and Table 4). Common activated signaling pathways between both PS particles included neuroinflammation, hepatic fibrosis, wound healing, and Rho family GTPases. Many of the activated pathways were involved in inflammation and cytoskeleton regulation. These pathways are involved in the activation of astrocytes through proliferation, migration/motility, changes in the cytoskeleton (changes in actin-assembly), and re-organization of the extracellular matrix (ECM). Activation of astrocytes is regulated by cytokines, chemokines, growth factors, and products of oxidative stress. Of note, both dose groups showed an overwhelming inflammatory response in our IPA pathway analysis.

There were two common inhibited pathways in both PS particles sizes, peroxisome proliferator-activated receptor (PPAR) signaling and Rho GDP-dissociation inhibitor (RHOGDI) signaling (Table 3 and Table 4). The 500 nm exposed astrocytes had an additional three unique pathways inhibited, most involved in lipid metabolism.

### 3.4. Predicted Activated and Inhibited Regulators

IPA identified 503 activated and 177 inhibited upstream regulators in the 50 nm PS particle exposed group. The 500 nm exposed group had 633 activated and 269 inhibited upstream regulators (Figure 6B). Interestingly, the top seven activated upstream regulators were common to both PS particles sizes (Table 5), while six out of ten inhibited upstream regulators were common between both groups (Table 6). The activated upstream regulators were mainly involved in pro-inflammatory pathways, while inhibitors of MAPK pathway U0126, PD98059, SB203580 were amongst the most significantly inhibited upstream regulators in both particle sizes.

### 3.5. GO Term Enrichment and KEGG Analysis

**Figure 7:**
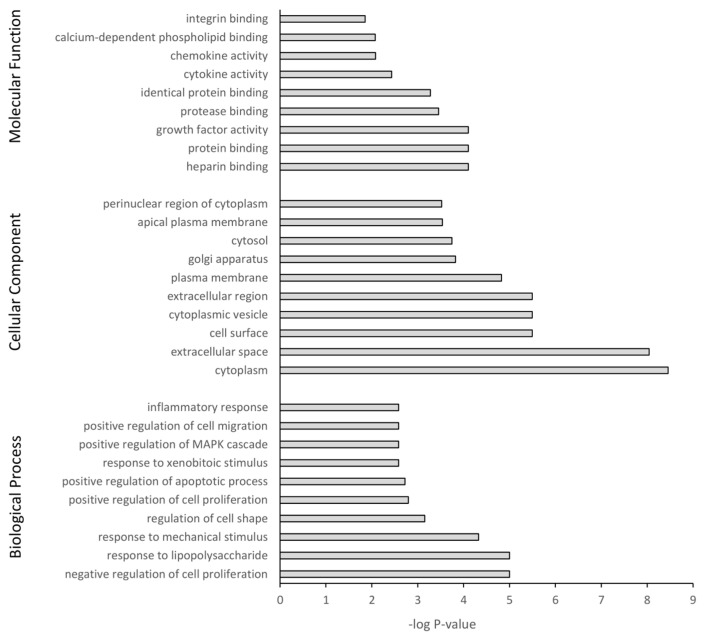
Gene ontology classification of genes differentially expressed between 50 nm PS-NP exposed astrocytes and untreated controls using DAVID. Benjamini adjusted *p*-value < 0.05.

**Figure 8:**
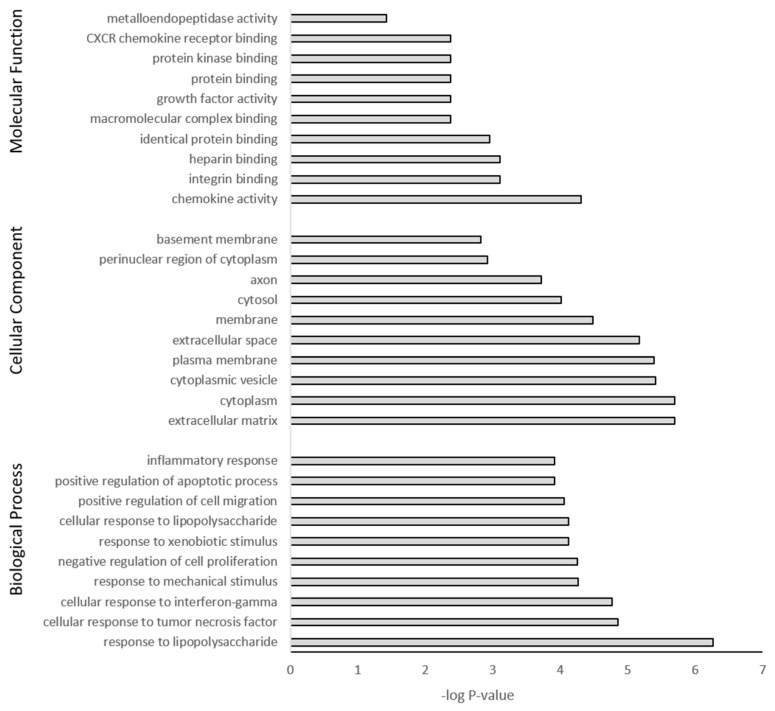
Gene ontology classification of genes differentially expressed between 500 nm PS-MP exposed astrocytes and untreated controls using DAVID. Benjamini adjusted *p*-value < 0.05.

The DEGs were further subjected to GO and KEGG pathway analysis. The top 10 pathway enrichments for three categories, namely biological process, cellular component, and molecular function, were determined by GO analysis (filtered at adjusted *p*-value < 0.05) for each PS particle size (50 nm Figure 7 and 500 nm Figure 8). Although each PS particle size generated unique pathway enrichment, they shared many GO terms, 19 out of 30. The common terms for biological process between 50 nm and 500 nm groups were response to lipopolysaccharide, response to mechanical stimulus, negative regulation of cell proliferation, response to xenobiotic stimulus, positive regulation of cell migration, positive regulation of apoptotic process and inflammatory response. Cellular component common terms were cytoplasm, extracellular space, cytoplasmic vesicle, plasma membrane, cytosol and perinuclear region of cytoplasm. The terms for molecular function shared between particle sizes were chemokine activity, integrin binding, heparin binding, identical protein binding, growth factor activity and protein binding (Supplementary Materials Tables S1 and S2). The 50 nm exposed group had no significant GO terms associated with the downregulated genes; however, the 500 nm group had five GO terms associated with the downregulated genes, with four of five related to the cell membrane (Supplementary Materials Table S2).

The top 10 enriched KEGG pathways for the 50 nm PS particle exposed group were MAPK signaling, apoptosis, cytokine-cytokine receptor interaction, NF-kappa B signaling, NOD-like receptor signaling, cellular senescence, IL-17 signaling, TNF signaling, p53 signaling and lipid and atherosclerosis (Table 7). For the 500 nm group, the top 10 enriched KEGG pathways were TNF signaling, IL-17 signaling, focal adhesion, MAPK signaling, NOD-like receptor signaling, p53 signaling, cytokine-cytokine receptor interaction, apoptosis, NF-κB signaling and chemokine signaling (Table 8). The two different PS-bead sizes elicited a very similar cellular response via upregulation of the same pathways, with 8 out of 10 pathways common between both PS-bead sizes. Furthermore, 10 out of the 12 unique KEGG pathways were involved in inflammation. In the 50 nm exposed group, no KEGG pathways corresponded with the downregulated genes, while the 500 nm exposed group had four, with two of the four involved in metabolism. Overall, pathway enrichment analysis revealed a common inflammatory cellular response to PS-MNPL exposure.

### 3.6. Distinct Pathways

Although there was a vast amount of similarity in the response to MPs and NPs in the astrocytes, there remained a subset of distinct differences. The 50 nm particles had a more cytotoxic effect, while the 500 nm particles caused more gene changes. In comparison, there were more pathways altered by exposure to 500 nm PS-microplastics compared to the 50 nm PS-nanoplastics. In IPA, there were 8 upregulated pathways unique to the 50 nm exposed astrocytes vs. control, and 38 unique upregulated pathways in the 500 nm vs. control. Some of the different upregulated pathways in the 500 nm group include HER-2 signaling in breast cancer, integrin signaling, NGF, EGF, and VEGF signaling. KEGG analysis showed enrichment for 1 unique pathway from the upregulated DEGs in the 50 nm, nitrogen metabolism, while 500 nm showed 26 distinctly different enriched pathways, which include PI3K-Akt, FoxO, HIF-1, Ras and JAK-STAT signaling pathways.

Only the 500 nm exposed astrocytes had distinct additional downregulated pathways. IPA revealed inhibited canonical pathways for LXR/RXR and PPARα/RXRα activation and apelin cardiac fibroblast signaling. KEGG analysis of downregulated DEGs indicated enrichment of metabolic pathways, amino acid degradation, GABAergic synapse and calcium signaling.

## 4. Discussion

There is a rising concern about the presence of nano- and microplastic pollution in the environment; however, their potential impact on human health needs further study. There have been several studies documenting the neurobehavioral and neurotoxic effects of MNPLs in aquatic organisms and mammals [37,56,57] and the presence of MNPLs have been detected in rodent brains upon exposure [3,17,39,40]. Currently, there are only a few studies that examine the cellular targets and molecular mechanisms of action of MNPLs in the brain. This study investigates the in vitro cellular response to PS particles of two different sizes (50 and 500 nm) at two time periods of exposure (24 h and 7 days) in the major neural cell types: neurons, astrocytes, oligodendrocytes, and neural stem cells. We monitored cell viability in all four cell types, as well as performed RNA-seq analysis on the astrocytes, as these cells were found to be the most susceptible to MNPL exposure. Overall, various brain cell types exposed to PS particles revealed that only astrocytes are highly impacted, and that, irrespective of PS particle size, an inflammatory response indicative of astrogliosis was induced.

### 4.1. Cytotoxicity

MTT assay revealed that after 24 h of PS particle exposure, only the astrocytes showed significant loss of cell viability at the highest dose, 1000 µg/mL. The other cell types showed minimal or no cytotoxicity at the doses tested. Similarly, other studies have observed comparable cytotoxic outcomes in the lower dose ranges tested. 1321N1 human astrocytoma cells treated with 50 nm pristine PS particles up to 100 µg/mL for 24 h showed no change in cell number or cell viability [58,59]. Primary astrocytes exposed to 100 nm PS particles up to 100 µg/mL for 48 h displayed no cell death [16]. However, 50 nm PS-NH2 particles decreased cell viability after 24 h in 1321N1 cells starting at 12.5 µg/mL [58,59,60]. Similarly, Murali et al. observed no cell death in neural stem cells (NE-4C), primary astrocytes, or stem cell-derived neurons and astrocytes upon exposure to 40–70 nm PS-COOH or PS-PEG particles up to 250 µg/mL after 24 h [41]. Lastly, T98G human glioblastoma cells showed no cytotoxicity when exposed to 40 nm, 250 nm or 10 µm PS particles up to 10 µg/mL for 48 h [61].

There is a distinct lack of studies investigating the long-term exposure of micro- and nanoplastics in vitro [62]. Since polystyrene particles are not biodegradable and can remain inside the cell, in addition to accumulation through continuous exposure, this could cause cellular stress and induce cytotoxicity by disrupting cellular functions, especially in long term cultures [16,23,62]. We examined cytotoxicity after chronic repeat exposure of 7 days. Interestingly, PS-MNPLs induced different levels of cytotoxicity in the various neural cells. We observed mild cytotoxicity in the neural stem cells and neurons that were exposed to 500 nm PS particles, with toxicity only observed at the highest dose of 1000 µg/mL and no cytotoxicity with the 50 nm PS particles. Oligodendrocytes showed moderate loss of cell viability when exposed to 500 nm PS particles > 100 µg/mL and no change with the 50 nm PS particles. Lastly, the astrocytes showed the greatest cytotoxicity, with cell viability decreasing at >10 µg/mL for 50 nm and remaining at 1000 µg/mL for 500 nm particles. In comparison, Hua et al. have observed that hiPSC-derived forebrain cortical spheroids have reduced cell viability when exposed to 1 µm MPs for 26 days [23]. Furthermore, other studies have noted a cell-specific response to MNPLs both in vivo and in vitro [16,17,41]. The differences in MNPL toxicity observed across studies may be a result of exposure duration, particle size, and cell type.

### 4.2. Internalization

Astrocytes showed the greatest response to MNPL exposure, with increased cytotoxicity after long term exposure. Subsequently, we examined the internalization of MNPLs by astrocytes and their parental cell line, neural stem cells (NSC). After 24 h exposure, MNPLs were efficiently taken up by both cell types. Fluorescent particles were detected inside the cell at the lowest concentration tested, reaching saturation by 10 ug/mL for the 50 nm PS-NPs and 100 µg/mL for the 500 nm PS-MPs. These results are in line with previous studies which have detected in vitro internalization of MNPLs in neural cells. 13121N1 human astrocytoma cells are able to internalize 20 nm, 40 nm, 100 nm and 200 nm carboxylated-PS particles and 50 nm amine-PS particles [60,63,64]. Primary astrocytes took up 100 nm PS particles in a concentration-dependent manner [16]. Similarly, Hua et al. observed internalization of 1 µm PS-MPs in hiPSC-derived cortical spheroid in a concentration-dependent manner [23]. However, in a primary brain culture containing neurons, astrocytes, and microglia exposed to PS-COOH NPs (40–70 nm), uptake was only noted in microglial cells [41]. Although both particle sizes (50 nm and 500 nm) were readily internalized, there appeared to be a difference in the percentage of FITC+ cells between particle sizes and cell type, suggesting different modes of internalization. The internalization of MNPLs can differ by particle size and cell type and act through a variety of mechanisms including clathrin/caveolae-mediated endocytosis and passive diffusion [65].

### 4.3. RNA-Seq Analysis

Astrocytes are involved in the control of immune cell activation and trafficking, as they are able to detect danger signals, secrete cytokines and chemokines, and activate the immune system [49,50]. Since we observed cytotoxicity and cellular uptake of MNPLs in astrocytes, we next investigated the changes in the transcriptome of astrocytes exposed for 7 days to MNPLs through RNA-seq. Given that the cytotoxicity was greater than 80% at 1000 µg/mL, we sequenced astrocytes exposed to MNPLs at 0, 1, 10 and 100 µg/mL exposure to PS particles. Our results showed that significant gene changes were only present at the 100 µg/mL dose. The 50 nm and 500 nm PS particle treatment of astrocytes mostly resulted in the overexpression of genes and there were more pathways activated than inhibited by the exposure to MNPLs. Of the top 10 activated pathways through IPA analysis, 63% were involved in inflammation; whereas overall, 50 nm particles had ~40% of activated pathways involved in inflammation or immune response, while 500 nm had ~34%. Furthermore, KEGG pathway analysis of upregulated DEGs corroborated this central axis of inflammatory gene expression to PS particle exposure with 83% of the unique top 10 upregulated pathways involved in inflammation. This inflammatory response is indicative of astrogliosis.

Astrocytes can respond to CNS trauma with changes in gene expression, cellular structure and function, in a reaction known as astrogliosis or reactive astrocytosis [48,66,67]. Astrogliosis encompasses a diverse and heterogeneous response, with complex and numerous gene changes, which are induced in a context-dependent manner [48,49,66]. In this activated state, astrocytes can secrete nitric oxide, prostaglandins, adhesion molecules, extracellular matrix proteins, amino acids, and cytokines [47,68]. As part of the inflammatory response, astrocytes are also able to form physical and molecular barriers (glial scars) that are able to seal the injury site and localize the neuroinflammatory response [69]. Overall, this process is complex and may include morphological changes/hypertrophy, migration, proliferation, and metabolic and transcriptional changes [48,49,66,69,70].

In our data, we have several upregulated genes that correspond with molecular signals released from reactive astrocytes, and these genes include *Lcn2*, *Il-11*, *Cxcl10*, *Ccl2*, *Ccl7*, *Cxcl1*, *Lif*, and *Ngf* [49,66]. Of note, we did not see an increase in glial fibrillary protein (*Gfap*) expression, a common marker of astrogliosis. However, as noted by Hamby et al., select inflammatory signals can markedly alter reactive astrocyte transcriptomes without inducing *GFAP* [67,71]. Also, GFAP expression varies with the type of injury [72]. It is not well understood how proinflammatory cytokine production and astrogliosis is induced by MNPLs and further study is needed to elucidate the mechanism.

#### 4.3.1. Inflammation

IPA and DAVID analysis of DEGs, KEGG pathways, and GO terms all suggest that PS-MNPL exposure led to changes characteristic of reactive astrocytes. Astrocytes express a variety of receptors, including pattern recognition receptors, which allow them to monitor their environment. Many of these receptors detect molecules occurring in pathological conditions, pathogen-associated molecular patterns/damage-associated molecular patterns (PAMPS/DAMPS) and cytokines [73]. Signaling of these receptors converge on one major pathway, the nuclear factor kappa-light-chain-enhancer of activated B cells (NF-κB) signaling pathway, which is a master regulator of inflammation and controls the pathophysiological response in astrocytes [74]. In our data, NF-κB is a predicted activated upstream regulator and several canonical pathways upregulated by PS particle exposure are involved in the inflammatory response including neuroinflammation, wound healing, acute phase response, high mobility group box 1 protein (HMGB1), interleukin (IL)-6 and IL-17. Neuroinflammation, one of the top pathways for both 50 nm and 500 nm exposed astrocytes involves the activation of innate immune cells, release of inflammatory mediators, such as cytokines and chemokines, and the generation of reactive oxygen species and nitrogen species [75,76]. IL-17 enhances IL-6 signaling and subsequently enhances further IL-6 expression, both of which were activated in IPA analysis, and IL-17 was an enriched KEGG pathway through DAVID analysis in response to PS particle exposure. IL-6 is a pro-inflammatory cytokine and is also a major inducer of acute phase proteins [77]. IL-17 also activates NF-κB and MAPK signaling pathways [77], both of which were enriched KEGG pathways. The NF-κB signaling cascade mediates production of pro-inflammatory cytokines, chemokines, and inducible enzymes nitric oxide synthase (iNOS) and COX-2, which all contribute to neuroinflammation [78,79,80]. The latter is a protective response by the brain to remove invading pathogens, neutralize noxious stimuli, and initiate tissue repair.

MNPLs are known to cause membrane stress and permeate the lipid membrane, which could lead to transient membrane permeabilization and release of DAMPs, ions, cytokines, and other molecules into the extracellular space [81]. Our data showed that HMGB1 signaling was an activated canonical pathway in both PS particle size exposure. HMGB1 is a DAMP molecule released into the extracellular space where it interacts with toll-like receptors and receptors for advanced glycation end-products, leading to the activation of mitogen-activated protein kinase (MAPK) pathways [82,83,84]. The MAPK pathways include the extracellular signal regulated kinase (ERK) pathway, the p38 pathway, and the C-Jun N-terminal kinases (JNK) pathway, and these pathways orchestrate production of inflammatory cytokines, extracellular matrix (ECM) proteins, and upregulates iNOS expression [73]. MAPK signaling was upregulated in the canonical pathways analysis and enriched in the KEGG analysis of exposure to both sizes of PS-MNPLs.

Furthermore, in the upregulated DEGs, several cytokines and chemokines were listed. *Ccl2*, *Ccl7*, *Cxcl10*, *Cxcl11* were commonly upregulated in exposure to both PS sizes, whereas 500 nm had a few more cytokines and chemokines upregulated in the top 10 (*Cxcl13* and *Ccl5*). Ccl2 and Cxcl10 are pro-inflammatory cytokines released by astrocytes upon activation of NF-κB and are associated with astrocyte reactivity, acting as important mediators of inflammation and play a major role in the recruitment of inflammatory cells [85]. In addition, these recruited cells can release numerous cytokines, which further stimulate astrocyte activation. Similar signaling has been observed with microglia cells, which can further induce astrogliosis via expression of pro-inflammatory cytokines, such as IL-1B [86].

Our results show a central theme of inflammation and inflammatory response to PS exposure, one of the hallmarks of astrogliosis, through the activation of NF-κB, MAPK, HMGB1, IL-17 and IL-6 signaling. The other characteristics of astrogliosis include morphological changes/hypertrophy, migration (scar formation), proliferation, ECMs and cytoskeleton remodeling, cellular senescence/apoptosis were also supported by the DEGs, IPA and KEGG pathway analysis, and GO terms.

#### 4.3.2. Cytoskeleton and Cell Migration

After inflammation, IPA analysis revealed that the next largest group of gene changes in response to PS-MNPL exposure is remodeling of the cytoskeletal/ECM, with 70% of the unique top 10 activate canonical pathways involved in cytoskeletal/ECM remodeling. In our data set, cardiac hypertrophy, hepatic fibrosis and wound healing were activated canonical pathways. These pathways share commonalities including cell morphology/hypertrophy, proliferation, reorganization of the ECM and cytoskeleton [87]. Cell migration, focal adhesion and remodeling of the ECM are fundamental to tissue morphogenesis, wound healing, hepatic fibrosis, and immune responses [88,89].

In this study, although no morphological changes were noted under the light microscope, GO terms were enriched for cell migration and cell shape. KEGG analysis revealed that the focal adhesion (FA) pathway was enriched. Focal adhesions are multi-protein complexes that anchor matrix receptors of the integrin family to the actin cytoskeleton [90]. The molecular function integrin binding was an enriched GO term in both 50 nm and 500 nm PS exposure, while the canonical pathway integrin signaling was activated in 500 nm only. In astrocytes, integrin-mediated signaling is known to govern cell spreading, morphology, migration and proliferation, all hallmarks of astrogliosis [91,92].

The GO term biological processes positive regulation of cell migration and negative and positive regulation of cell proliferation were observed in both sizes of PS particles tested. Furthermore, cell migration requires coordinated changes to the cytoskeleton [93]. In canonical pathway analysis, we note that signaling by Rho family GTPases, RHOA signaling and RAC signaling were activated, while RHOGDI signaling, a negative regulator of the Rho family, was inhibited. The small GTPases of the Rho family are involved in cellular polarity [94]. RhoA signaling is active towards the rear of the cell, regulating its retraction during migration, whereas Rac1 signaling is active towards the front of the cell [95]. During astrogliosis, astrocytes are known to change their morphology from a more round-like aspect to elongated shapes, to reorganize their arborization and the number of processes and/or polarize towards the injury site and surround necrotic and inflamed tissue [96,97]. Further, these astrocytes retract their processes, become polarized and acquire front-to-rear asymmetry typical of migratory cells [98].

#### 4.3.3. Ubiquitin Proteasome System

The highest upregulated DEG in both 50 nm and 500 nm exposed astrocyte groups is Ubiquitin D (*Ubd*, also called FAT10). The ubiquitin proteasome system (UPS) uses ubiquitination to select target proteins for degradation [99]. Ubiquitin signaling also plays a critical role in homeostasis through receptor trafficking, DNA damage response, mitophagy, and inflammation [100]. Ubiquitin D directly targets proteins for proteasomal degradation and is induced by pro-inflammatory cytokines interferon gamma (IFN-γ) and tumor necrosis factor (TNF-α) [101], both of which are activated upstream regulators from the IPA analysis. Canonical pathway analysis also revealed FAT10 cancer signaling pathway as activated in the top 20 pathways for both 50 nm and 500 nm PS treated astrocytes.

Additionally, heat shock proteins (HSPs) act as molecular chaperones and are involved in protein folding and they direct misfolded proteins for degradation [102]. *Hspb1* is the most highly upregulated heat shock protein gene in our data set. It is abundant in astrocytes under normal physiological conditions, but is elevated in response to stress conditions and is thought to play a role in astrogliosis [103]. Moreover, HSPB1 is able to regulate the activation of the NF-κB pathway, resulting in a pro-inflammatory response [103]. In addition to the upregulation of *Hspb1*, *Hspb8*, *Hspa1a* and *Hspa2*, HSP70 family members (1A and 2) were also upregulated in response to PS-NP and PS-MP treatment.

#### 4.3.4. p53 Signaling, Apoptosis, Cellular Senescence

DNA damage response pathways preserves genomic integrity through a set of cellular responses, which include cell cycle control, DNA repair, and cell death [104,105], all controlled by the master regulator p53. KEGG pathway analysis showed that p53 signaling is enriched by PS particles. As a result of induced p53 signaling, we also observed enriched cellular senescence and apoptosis pathways.

In both PS particle sizes, there was an upregulation in the expression of growth arrest and DNA-damage-inducible genes belonging to the *Gadd45* family (*a*, *b* and *g*). Gadd45a encodes a ubiquitously expressed protein that is often induced by DNA damage and other stress signals associated with growth arrest [106]. Gadd45b and Gadd45g have been implicated in various responses to cell injury, including cell cycle checkpoints and apoptosis [106].

Apoptosis is the most widely known process of programmed cell death and is a defense mechanism involved in clearing damaged cells [107]. GO terms under biological processes also showed an enrichment for positive regulation of apoptosis for both the 50 nm and 500 nm exposed astrocytes. Additionally, studies in zebrafish show that PS-MP exposure induced dose- and time-dependent apoptotic responses [108]. In vivo, MPs were also shown to enhance apoptosis in cardiac rat tissue [40].

Cellular senescence can be triggered by numerous internal or external stressors including DNA damage, rapid replication, oxidative stress, mitochondria dysfunction, and proteasome dysfunction [69]. Senescence is permanent cell cycle arrest in the G1/G2 phase of the cell cycle and is p53-dependent [69]. Senescent astrocytes have increased expression of p21^waf1^ and p16^ink4a^, both involved in cell cycle arrest [69]. In both 50 nm and 500 nm exposed astrocytes, p21 (*Cdkn1a*) was upregulated, whereas only 500 nm PS particles upregulated p16 (*Cdkn2a*). Furthermore, in the 500 nm exposed group, in addition to the upregulation of *Gadd45a*, *Sfn* was also upregulated. Sfn (14-3-3-σ), a negative regulator of the cell cycle, inhibits the Cdc2-cyclin B1 complex, resulting in G2 arrest [109].

On top of permanent growth arrest, senescent astrocytes adopt a senescence-associated secretory phenotype (SASP) characterized by increased secretion of chemokines, growth factors, proteases, and inflammatory cytokines all under the control of HMGB1 [69,110]. Increased HMGB1 is considered the hallmark of senescent astrocytes [69] and the HMGB1 signaling canonical pathway was activated by both PS particles. The SASP secretome, which results from excessive senescence, alerts the immune system to the onset of injury, coordinates the deletion of senescent cells and can contribute to increased cancer risk and age-related diseases [110,111,112,113]. Studies have shown that exposure to MPs/NPs leads to increased cellular senescence, as seen in rat lung tissue, zebrafish gills, and porcine coronary artery endothelial cells exposed to PS-MPs [114,115,116].

#### 4.3.5. Downregulated Lipid Metabolism

Canonical pathway analysis showed two common downregulated pathways, PPAR and RHOGD1 signaling, in both 50 and 500 nm PS exposed astrocytes, though in the 500 nm treatment, a few additional pathways were observed, including LXR (liver X receptors)/RXR (retinoid X receptors), PPARα/RXRα activation, and apelin cardiac fibroblast signaling.

LXRs and PPARs are hormone nuclear receptors that form heterodimers with RXR to regulate lipid metabolism [117,118]. LXRs are master regulators of lipogenesis and cholesterol synthesis [117], whereas PPARs are key regulator of lipid and glucose homeostasis. PPARα and PPARγ are repressed via an NF-κB-dependent mechanism upon toll-like receptor (TLR) activation [119]. Our data showed that several upstream regulators connect to TLR activation, such as NF-κB, TNF-α, lipopolysaccharide, and MYD88 were all upregulated. Also, TLR signaling can reduce LXR activity, which functions to release the inhibition of NF-κB-dependent inflammatory gene expression [120].

Down-regulation of LXRs/RXRs and PPAR pathways suggest reduced cholesterol synthesis. Astrocytes synthesize and secrete cholesterol for neurons as the latter are unable to synthesize their own [121,122]. The DEGs in the 500 nm exposed group corresponding to altered lipid metabolism and cholesterol oxidation were sterol 27-hydroxylase (*Cyp27a1*), which was upregulated, whereas oxysterol 7-alpha-hydroxylase (*Cyp7b1*) and cholesterol-24-hydroxylase (*Cyp46a1*) were downregulated. Cholesterol can be oxidized in two ways; firstly, by 27-hydroxylase (*Cyp27a1*) to 27-hydroxycholesterol (27-OHC) and subsequently to 7α-hydroxy-3-oxo-4-cholestenoic acid (7-HOCA) by oxysterol 7-alpha-hydroxylase (*Cyp7b1)*. Since *Cyp7b1* was downregulated in 500 nm exposed astrocytes, further oxidation from 27-OHC to 7-HOCA may be limited, which suggests a buildup of 27-OHC. Secondly, cholesterol can also be oxidized to 24(S)-hydroxycholesterol (24-OHC) by cholesterol 24-hydroxylase (*Cyp46a1*), which was also downregulated in the 500 nm exposed astrocyte group. Our data indicated that there may have been an increased 27-OHC: 24-OHC ratio, which has been noted elsewhere to promote pro-inflammatory molecule release and neuroinflammation [123,124]. Furthermore, in the DAVID analysis of GO terms, functional annotation under biological process revealed that 28 of the downregulated DEGs were involved in lipid metabolism, of which 10 were specifically involved in fatty acid metabolism. These signaling pathways all contribute to the pro-inflammatory response induced in astrocytes exposed to PS particles.

This mechanistic study identified pathways affected in MNPL-exposed stem cell-derived astrocytes. A caveat of current research and its impact on risk assessment is the absence of standardized methods and analytical techniques for measuring nano- and microplastics in environmental matrices, including foods, as a result of which the extent and nature of human exposure are not well characterized. Potential human exposure may be dependent on exposure routes, particle size, surface chemistry, and particle composition. With no reports on how much environmental plastic exposure accumulates in the human body, through single exposure or cumulative exposure, and limited data available on the presence of MNPLs in the human diet [6], doses for in vitro work vary widely. The concentrations tested in this study ranged from 0.01 µg/mL to 1000 µg/mL and toxic effects were reported in the higher range, which may not be representative of anticipated human exposure to MNPLs. Further research is needed to determine environmentally relevant concentrations. Additionally, some uncertainties still remain in regard to potential exposure levels, as well as absorption of MNPLs and their distribution to various tissues and cell types in vivo. Consequently, some uncertainties remain in the extrapolation of in vitro mechanistic data to potential human health effects resulting from exposure to MNPLs.

## 5. Conclusions

There are growing concerns about the rise in the use of plastics and the subsequent presence of MNPLs in the environment, which highlight a need to evaluate the potential impact of MNPLs on human health. Recent studies have identified the potential for neurotoxicity, but the effects of MNPLs on the brain remain unclear. Our mechanistic results show that direct exposure of rat neural cell types to MNPL in vitro resulted in a diverse response, with astrocytes being the most sensitive to MNPL induced toxicity. Astrocytes were able to uptake the particles into the cytosolic space. Exposure to 500 nm PS-MPs induced a greater effect than to the 50 nm PS-NPs as observed by the number of DEGs changes and the magnitude of fold increases, but was less cytotoxic than the 50 nm NPs. At high levels of MNPL exposure, DEGs analyzed through IPA and DAVID revealed that both particle sizes lead to the upregulation or enrichment of pathways involved in reactive astrocytes including inflammation, immune response, migration, proliferation, ER-stress related pathways and proteasomal dysfunction, cell-death pathways, and remodeling of the cytoskeleton and ECM. The downregulated pathways were related to lipid metabolism. Our study contributes to the understanding of the mechanism of neurotoxicity of MNPLs in mammalian in vitro models by elucidating the impact of MNPLs on NSCs and different neural cell types.

## Figures and Tables

**Table 1:** Top 10 Up- and downregulated DEGs in astrocytes exposed to 50 nm PS-NPs.

Gene Symbol	Gene Annotation	Fold Change	Adjusted *p*-Value
**Ubd**	Ubiquitin D	597.1	8.90 × 10^−4^
Mir675	Micro RNA 675	171.8	2.74 × 10^−7^
**Ccl7**	C-C Motif Chemokine Ligand 7	70.8	2.53 × 10^−7^
**Ccl2**	C-C Motif Chemokine Ligand 2	69.4	1.44 × 10^−13^
Ifit3	Interferon Induced Protein with Tetratricopeptide Repeats 3	53	1.97 × 10^−4^
Aldh1a3	Aldehyde Dehydrogenase 1 Family Member A3	39.6	3.61 × 10^−5^
Fgf10	Fibroblast Growth Factor 10	37.5	1.34 × 10^−4^
**Slfn2**	Schlafen Family Member 12 Like	35.7	9.08 × 10^−5^
Cxcl10	C-X-C Motif Chemokine Ligand 10	35.1	4.15 × 10^−6^
**Cxcl11**	C-X-C Motif Chemokine Ligand 11	26.5	1.90 × 10^−3^
Scube2	Signal Peptide, CUB Domain And EGF Like Domain Containing 2	−2.7	6.80 × 10^−5^
Veph1	Ventricular Zone Expressed PH Domain Containing 1	−2.8	1.94 × 10^−4^
Cfap100	Cilia And Flagella Associated Protein 100	−3	3.07 × 10^−3^
Psd4	Pleckstrin And Sec7 Domain Containing 4	−3	1.21 × 10^−4^
**Slc27a5**	Solute Carrier Family 27 Member 5	−3.1	3.33 × 10^−4^
**Mroh7**	Maestro Heat Like Repeat Family Member 7	−3.3	6.27 × 10^−3^
Fgfr4	Fibroblast Growth Factor Receptor 4	−3.3	2.29 × 10^−3^
Kcng1	Potassium Voltage-Gated Channel Modifier Subfamily G Member1	−3.7	9.19 × 10^−4^
Nmb	Neuromedin B	−4.1	6.34 × 10^−3^
**Fmo1**	Flavin Containing Dimethylaniline Monooxygenase 1	−4.3	2.45 × 10^−8^

Note: The common DEGs between 50 nm and 500 nm treated astrocytes are highlighted in bold.

**Table 2:** Top 10 Up- and downregulated DEGs in astrocytes exposed to 500 nm PS-MPs.

Gene Symbol	Gene Annotation	Fold Change	Adjusted *p*-Value
**Ubd**	Ubiquitin D	3909.9	1.15 × 10^−8^
Cxcl3	C-X-C Motif Chemokine Ligand 3	1036.1	6.82 × 10^−5^
Ccl5	C-C Motif Chemokine Ligand 5	403.2	7.09 × 10^−9^
**Ccl2**	C-C Motif Chemokine Ligand 2	242.1	3.39 × 10^−9^
**Ccl7**	C-C Motif Chemokine Ligand 7	152.9	1.94 × 10^−9^
Tfpi2	Tissue Factor Pathway Inhibitor 2	127.6	2.86 × 10^−3^
Trabd2b	TraB Domain Containing 2B	109.8	5.87 × 10^−6^
Atp6v0d2	ATPase H+ Transporting V0 Subunit D2	93.7	1.76 × 10^−3^
**Slfn2**	Schlafen Family Member 12 Like	93.3	1.05 × 10^−7^
**Cxcl1**	C-X-C Motif Chemokine Ligand 1	88	5.98 × 10^−8^
Dcdc2	Doublecortin Domain Containing 2	−7.8	1.50 × 10^−5^
P2rx6	Purinergic Receptor P2X 6	−8.6	1.05 × 10^−18^
Gm45623	Small Integral Membrane Protein 32 (Smim32)	−9.2	7.02 × 10^−5^
Trpm5	Transient Receptor Potential Cation Channel Subfamily M Member 5	−9.6	6.22 × 10^−4^
**Slc27a5**	Solute Carrier Family 27 Member 5	−11.3	4.56 × 10^−9^
**Fmo1**	Flavin Containing Dimethylaniline Monooxygenase 1	−12	4.60 × 10^−13^
LOC689725	Open Reading Frame (ORF)	−14.1	9.19 × 10^−4^
Ankfn1	Ankyrin Repeat and Fibronectin Type III Domain Containing 1	−14.5	1.72 × 10^−8^
**Mroh7**	Maestro Heat Like Repeat Family Member 7	−15.6	2.64 × 10^−5^
Fmo2	Flavin Containing Dimethylaniline Monooxygenase 2	−24.5	1.96 × 10^−3^

Note: The common DEGs between 50 nm and 500 nm treated astrocytes are highlighted in bold.

**Table 3:** Activated and inhibited canonical pathways for 50 nm NP treated astrocytes.

**Activated Canonical Pathway**	**Z-Score**	**−log *p*-Value**
Cardiac Hypertrophy Signaling (Enhanced)	3.8	4.65
**Signaling by Rho Family GTPases**	3.77	5.53
**Hepatic Fibrosis Signaling Pathway**	3.67	6.16
**Neuroinflammation Signaling Pathway**	3.55	7.36
PI3K Signaling in B Lymphocytes	3.46	4.41
Cardiac Hypertrophy Signaling	3.16	1.72
Acute Phase Response Signaling	3.15	7.29
**Wound Healing Signaling Pathway**	3.13	6.56
RHOA Signaling	3	3.63
Role of NFAT in Cardiac Hypertrophy	3	2.21
**Inhibited Canonical Pathway**	**Z-Score**	**−log *p*-Value**
**PPAR Signaling**	−2.33	3.46
**RHOGDI Signaling**	−2.11	3.30

Note: The common canonical pathways between 50 nm and 500 nm treated astrocytes are highlighted in bold.

**Table 4:** Activated and inhibited canonical pathways for 500 nm MP treated astrocytes.

**Activated Canonical Pathway**	**Z-Score**	**−log *p*-Value**
**Neuroinflammation Signaling Pathway**	4.16	7.06
**Signaling by Rho Family GTPases**	3.92	7.04
**Wound Healing Signaling Pathway**	3.78	4.43
HMGB1 Signaling	3.64	4.73
HER-2 Signaling in Breast Cancer	3.58	2.14
**Hepatic Fibrosis Signaling Pathway**	3.57	7.03
IL-13 Signaling Pathway	3.5	3.84
IL-6 Signaling	3.5	4.36
Role of Hypercytokinemia/Hyperchemokinemia in the Pathogenesis of Influenza	3.46	3.07
RAC Signaling	3.46	3.92
**Inhibited Canonical Pathway**	**Z-Score**	**−log *p*-Value**
LXR/RXR Activation	−2.71	2.61
PPARα/RXRα Activation	−2.67	2.53
**PPAR Signaling**	−2.50	3.72
**RHOGDI Signaling**	−2.14	3.12
Apelin Cardiac Fibroblast Signaling Pathway	−2	1.64

Note: The common canonical pathways between 50 nm and 500 nm treated astrocytes are highlighted in bold.

**Table 5:** Top 10 upstream regulators activated by 50 nm and 500 nm PS-MNPLs.

50 nm	Z-Score	−log *p*-Value	500 nm	Z-Score	−log *p*-Value
**Lipopolysaccharide**	9.33	32.48	**Lipopolysaccharide**	9.94	33.17
**TNF**	7.68	43.67	**TNF**	8.58	44.88
**IL1B**	7.46	38.28	**poly rI:rC-RNA**	7.70	11.98
**IFNG**	7	41.36	**IL1B**	7.67	32.25
**poly rI:rC-RNA**	6.90	23.02	**tetradecanoylphorbol acetate**	7.67	23.94
**NFkB (complex)**	6.78	31.96	**NFkB (complex)**	7.46	25.81
**tetradecanoylphorbol acetate**	6.74	22.83	**IFNG**	6.78	31.81
IL6	6.26	23.43	F2	6.55	21.78
Cisplatin	5.76	17.26	KLF6	6.37	21.29
*E. coli* B5 lipopolysaccharide	5.75	20.55	MYD88	6.27	12.40

Note: The upstream regulators common in both 50 nm and 500 nm exposed astrocytes are highlighted in bold.

**Table 6:** Top 10 upstream regulators inhibited by 50 nm and 500 nm PS-MNPLs.

50 nm	Z-Score	−log *p*-Value	500 nm	Z-Score	−log *p*-Value
**U0126**	−5.59	20.67	**U0126**	−6.40	21.40
**SB203580**	−5.53	18.75	**PD98059**	−5.94	21.83
**LY294002**	−5.35	19.12	**SB203580**	−5.60	18.21
**PD98059**	−5.25	24.29	**CITED2**	−5.56	7.28
**CITED2**	−5.01	12.10	SP600125	−5.54	17.59
TSC2	−4.78	12.71	**LY294002**	−5.25	18.41
**Alpha catenin**	−4.26	13.62	**Alpha catenin**	−4.52	13.16
TREX1	−4.23	15.27	Irgm1	−4.40	7.07
N-acetyl-l-cysteine	−4.10	8.77	SIRT1	−4.39	8.75
SN-011	−3.71	9.52	SB-431542	−4.32	8.25

Note: The upstream regulators common in both 50 nm and 500 nm exposed astrocytes are highlighted in bold.

**Table 7:** The 50 nm KEGG pathways.

KEGG Pathways Associated with Upregulated Genes	Count	%	Adjusted *p*-Value
**MAPK signaling pathway**	24	6	7.90 × 10^−5^
**Apoptosis**	16	4	7.90 × 10^−5^
**Cytokine-cytokine receptor interaction**	22	5.5	1.00 × 10^−4^
**NF-kappa B signaling pathway**	13	3.2	1.30 × 10^−4^
**NOD-like receptor signaling pathway**	17	4.2	1.50 × 10^−4^
Cellular senescence	16	4	8.00 × 10^−4^
**IL-17 signaling pathway**	11	2.7	1.20 × 10^−3^
**TNF signaling pathway**	11	2.7	4.70 × 10^−3^
**p53 signaling pathway**	9	2.2	4.70 × 10^−3^
Lipid and atherosclerosis	15	3.7	5.60 × 10^−3^

Note: The KEGG pathways common in both 50 nm and 500 nm exposed astrocytes are highlighted in bold.

**Table 8:** **The** 500 nm KEGG pathways.

**KEGG Pathways Associated with Upregulated Genes**	**Count**	**%**	**Adjusted *p*-Value**
**TNF signaling pathway**	23	3.1	9.20 × 10^−8^
**IL-17 signaling pathway**	19	2.6	2.40 × 10^−6^
Focal adhesion	27	3.7	5.20 × 10^−6^
**MAPK signaling pathway**	33	4.5	8.90 × 10^−6^
**NOD-like receptor signaling pathway**	24	3.3	2.30 × 10^−5^
**p53 signaling pathway**	15	2	3.00 × 10^−5^
**Cytokine-cytokine receptor interaction**	30	4.1	3.00 × 10^−5^
**Apoptosis**	20	2.7	4.90 × 10^−5^
**NF-kappa B signaling pathway**	16	2.2	1.50 × 10^−4^
Chemokine signaling pathway	22	3	2.10 × 10^−4^
**KEGG Pathways Associated with Downregulated Genes**	**Count**	**%**	**Adjusted *p*-Value**
Metabolic pathways	68	14.5	1.00 × 10^−5^
Valine, leucine and isoleucine degradation	9	1.9	2.50 × 10^−3^
GABAergic synapse	11	2.4	2.50 × 10^−3^
Calcium signaling pathway	16	3.4	1.50 × 10^−2^

Note: The KEGG pathways common in both 50 nm and 500 nm exposed astrocytes are highlighted in bold.

## Data Availability

Raw datasets can be accessed through GSE256038 for the NP 50 nm and MP 500 nm RNA-sequencing datasets.

## Supplementary Materials

The following supporting information can be downloaded at: https://www.mdpi.com/article/10.3390/nano14050429/s1, Table S1: The 50 nm GO terms; Table S2: The 500 nm GO terms; Table S3: Dynamic light scattering PS-NP and PS-MP characterization.
