# Supplementary material for: Polystyrene Nano- and Microplastic Particles Induce an Inflammatory Gene Expression Profile in Rat Neural Stem Cell-Derived Astrocytes In Vitro
Source: Nanomaterials (Basel). 2024 Feb 27;14(5):429. doi: 10.3390/nano14050429 (PMC10935329; doi:10.3390/nano14050429)
Supplement: Supplementary file 1 [file nanomaterials-14-00429-s001.zip › nanomaterials-2865258-supplementary.pdf]

Supplemental Table S1: 50nm GO terms

Upregulated	Term	Count	%	P-Value
Biological Process	negative regulation of cell proliferation	31	7.7	1.00E-05
	response to lipopolysaccharide	24	6	1.00E-05
	response to mechanical stimulus	15	3.7	4.70E-05
	regulation of cell shape	15	3.7	7.00E-04
	positive regulation of cell proliferation	17	4.2	1.60E-03
	positive regulation of apoptotic process	24	6	1.90E-03
	response to xenobiotic stimulus	28	6.9	2.60E-03
	positive regulation of MAPK cascade	16	4	2.60E-03
	positive regulation of cell migration	19	4.7	2.60E-03
	inflammatory response	21	5.2	2.60E-03
Cellular Component	Cytoplasm	172	42.7	3.50E-09
	extracellular space	72	17.9	9.10E-09
	cell surface	39	9.7	3.20E-06
	cytoplasmic vesicle	27	6.7	3.20E-06
	extracellular region	44	10.9	3.20E-06
	plasma membrane	133	33	1.50E-05
	golgi apparatus	46	11.4	1.50E-04
	Cytosol	112	27.8	1.80E-04
	apical plasma membrane	24	6	2.90E-04
	perinuclear region of cytoplasm	35	8.7	3.00E-04
Molecular Function	heparin binding	17	4.2	7.90E-05
	protein binding	67	16.6	7.90E-05
	growth factor activity	16	4	7.90E-05
	protease binding	14	3.5	3.50E-04
	identical protein binding	68	16.9	5.30E-04
	cytokine activity	14	3.5	3.70E-03
	chemokine activity	7	1.7	8.30E-03
	calcium-dependent phospholipid binding	8	2	8.40E-03
	integrin binding	12	3	1.40E-02

Supplemental Table S2: 500nm GO terms

Upregulated	Term	Count	%	P-Value
Biological Process	response to lipopolysaccharide	36	4.9	5.40E-07
	cellular response to tumor necrosis factor	26	3.6	1.40E-05
	cellular response to interferon-gamma	20	2.7	1.70E-05
	response to mechanical stimulus	19	2.6	5.50E-05
	negative regulation of cell proliferation	41	5.6	5.60E-05
	response to xenobiotic stimulus	45	6.1	7.50E-05
	cellular response to lipopolysaccharide	31	4.2	7.50E-05
	positive regulation of cell migration	30	4.1	8.70E-05
	positive regulation of apoptotic process	37	5.1	1.20E-04
	inflammatory response	33	4.5	1.20E-04
Cellular Component	extracellular matrix	33	4.5	2.00E-06
	cytoplasm	265	36.2	2.00E-06
	cytoplasmic vesicle	38	5.2	3.90E-06
	plasma membrane	221	30.2	4.10E-06
	extracellular space	98	13.4	6.70E-06
	membrane	62	8.5	3.30E-05
	cytosol	52	7.1	9.60E-05
	axon	93	12.7	1.90E-04
	perinuclear region of cytoplasm	178	24.3	1.20E-03
	basement membrane	37	5.1	1.50E-03
Molecular Function	chemokine activity	12	1.6	4.90E-05
	integrin binding	20	2.7	7.90E-04
	heparin binding	21	2.9	7.90E-04
	identical protein binding	107	14.6	1.10E-03
	macromolecular complex binding	47	6.4	4.20E-03
	growth factor activity	18	2.5	4.20E-03
	protein binding	96	13.1	4.20E-03
	protein kinase binding	41	5.6	4.20E-03
	CXCR chemokine receptor binding	6	0.8	4.20E-03
	metalloendopeptidase activity	14	1.9	3.80E-02

Downregulated	Term	Count	%	P-Value
Cellular Component	axonemal dynein complex	5	1.1	1.90E-03
	integral component of presynaptic membrane	11	2.4	2.60E-03
	integral component of postsynaptic membrane	10	2.1	3.40E-03
	membrane	54	11.5	3.30E-02
	receptor complex	14	3	3.80E-02

Supplemental Table S3: PS-NP and -MP characterization

	NPs		MPs	
	Distilled H2O	Media	Distilled H2O	Media
Size (nm)	78.20 ± 0.67	87.21 ± 0.17	494.9 ± 5.9	546.97 ± 8.05
PdI	0.19 ± 0.004	0.09 ± 0.01	0.04 ± 0.005	0.1 ± 0.02

Methods:

Fluoresbrite YG beads were diluted 1:300 in distilled water or astrocytes complete media (DMEM). Plastics hydrodynamic size was characterized by dynamic light scattering (DLS) using a Zetasizer (PN3702, Malvern, UK).
